# The Hospital Nursing Supplement

**Published:** 1893-03-25

**Authors:** 


### "The Hospital" Nursing Mirror.

Being the Extra Nursing Supplement of "The Hospital" Newspaper.

[Contributions for this Supplement should be addressed to the Editor, The Hospital, 428, Strand, London, W.C., and should have the word "Nursing" plainly written in left-hand top corner of the envelope.]

### News from the Nursing World.

### THE EAST END MOTHERS' HOME.

The East End Mothers' Home continues its useful work in its customary unpretentious way. The twelve beds are occupied, and heartily appreciated by the poor married women of the district. This modest little establishment is the only Maternity Home in all the vast East End of London, and it gives valuable training to midwives and nurses, for attendance on the poor in their own homes.

### OUR FISHERMEN.

There are many details of the medical and surgical work connected with the Deep Sea Mission of which but little is generally known. During last year, for instance, 8,000 men received medical treatment, and we find from the Society's magazine, the "Toilers of the Deep," that "when it was found that a few days on the hospital ship would pull a man round, the Society took him as an in-patient, and supplied a man to do his work." No wonder the hospital ships are appreciated by the fishermen, and we rejoice to hear that the city of Manchester has decided to support a vessel. We trust that other towns will follow this good example.

### HEALTH TALKS.

Miss Florence Nightingale's letter to village mothers can well be studied and appropriated by townsfolk too. Sanitary science is pleasant and attractive as seen with her eyes: "Boys and girls must grow up healthy, with clean minds and clean bodies and clean skins." And again Miss Nightingale banishes the solemn word "lecture," and substitutes the quaint title of "Homely Talk about Homes" as descriptive of the addresses of the lady lecturer whom she advises the hearers to make their friend.

### PRACTICAL LECTURING.

The syllabus of a course of lectures not infrequently causes dismay to the reader thereof. It often sets forth such a jumble of ill-arranged subjects that the mere names convey a foretaste of the confusion which will assuredly overtake the unwary aspirant in search of knowledge. With visions such as these constantly before us, Miss Catherine Wood's excellent syllabus of six "Lectures on Home Nursing" is quite a refreshing change from other leaflets. The programme should certainly attract large audiences to the course, wh'ch commences at 3.30 on April 7th at Fitzroy Hall.

### MORE COTS WANTED.

Perhaps the saddest word in our language is "incurable" when it is applied to a child. This, however, was not the chief impression given last week at the little Hospital for Incurable Children, 2, Maida Vale, which looked its best on the occasion of the last annual meeting. Fine weather prevailed on March 12th, and the bright sunshine showed off the spotless condition of the wards. Canon Duckworth, who had much to do with the first establishment of the hospital in 1875, took the chair, and showed unabated interest in the excellent work. A scheme is afloat for increasing the number of beds, and as this addition to the house can be made at the moderate cost of £500 to £700, we hope that lack of funds will not be permitted to cripple a most desirable movement. Homes for incurable and chronic cases are so few, and so inadequate to meet the demand for them, that everyone should be interested in securing more accommodation for these sorely afflicted children. The valuable aid given by the lady workers, whose privilege it is to help in the nursing and teaching of the little ones, was cordially acknowledged at the meeting, and the matron, Miss Coleman, was spoken of with hearty and affectionate appreciation on all sides, showing that the remark made by a gentleman respecting her was the universal opinion. He spoke of Miss Coleman as the mainstay, the chief prop and support of this successful institution, which works without the friction which unfortunately often clogs the machinery of many so-called charitable institutions.

### COMMON SENSE.

The House Committee of the Devon and Exeter Hospital gave a reply to the Council's question the other day, which many hospitals and nursing institutions will commend and copy. "That the committee cannot out of their number of nurses make a special roll for cholera cases, but that they are always ready to send nurses, if available, from their private nursing staff to infectious and contagious cases.

### MODERATE REMUNERATION.

Paragraphs, as well as various advertisements, are constantly bringing before us the urgent demand for trained nurses in workhouse infirmaries. If some of our readers were to visit the excellently planned wards of the modern institutions, they would speak less disparagingly of this branch of sick nursing. But our advocacy of workhouses is somewhat checked by the reports of the guardians' meetings which constantly claim our attention. At Thrapstone, for instance, we find the nurse, who is also matron's assistant, has worked well for three years at an annual salary of £18. "They had had in the past considerable difficulty with their officers, and it was satisfactory to note the harmony existing between them." The nurse, being apparently approved of on all sides, the guardians have agreed to raise her salary to the magnificent sum of £20 per annum. Even this will not leave her much chance of providing for possible sickness, nor yet for old age.

### GOOD HEALTH AND GOOD TEMPER.

How largely good temper depends upon a satisfactory condition of health is seldom acknowledged. After an exhibition of petulance a person commonly feels affronted by inquiries as to his bodily health, but mothers and nurses know that it is not only in nurseries that crossness is accounted for by physical ailments. There are noble examples amongst us of delicate people and even chronic invalids whose calm tempers are as admirable as their patience, but ordinarily in every day life we have less pleasant experiences. Dr. Welldon, Head Master of Harrow, gave an address to the Birmingham Teachers' Association the other day, and declared that "To be well is often the secret of teaching well." If we substitute the word "nursing" for "teaching," the truth is equally obvious. Dr. Welldon also said that good temper is essential to courtesy, and we must all acknowledge that courtesy is an essential in hospital life. This brings us again to our starting point, and it would be well if all workers strove earnestly to secure and to maintain that good health without which good temper can never be certainly reckoned upon.

### NURSES FOR NORFOLK.

A suggestion to supplement the staff of the West Norfolk and Lynn Hospital by additional trained nurses is now under consideration. There is evidently a demand for more private nurses in the neighbourhood, so the plan proposed should give general satisfaction, after it has been somewhat modified. As it stands at present, it can hardly commend itself to our readers. The Weekly Board decided, at a special meeting the other day, that two trained nurses should be engaged to go out private nursing, at from one to two guineas a week, and "that their salaries do not exceed £25." The plan is to be tried for six months, and a guarantee fund is to be formed "to protect the funds of the hospital from any possible deficiency." Thoroughly trained nurses, competent to take private cases, will hardly be content with £25 per annum, and there seems small fear that any "possible deficiency" can accrue to the funds of a hospital which reserves to itself so large a proportion of each private nurse's earnings.

### ACCOMMODATION FOR ACCIDENTS.

The need for a cottage hospital at Barry was discussed at the annual meeting of the District Nursing Association. Every one agreed as to the value of a place where local accidents could be treated, instead of, as at present, being taken all the way to Cardiff Infirmary. The Association proposes to again address the Local Board with regard to the necessity for erecting a small cottage hospital. Pending this arrangement, Dr. Powell suggested that a ward might be temporarily provided at the Nurses' Home, Barry Dock.

### A QUEEN'S NURSE FOR PEEBLES.

A public meeting was recently held at Peebles to discuss the need for a skilled district nurse. There seemed to exist no doubt in the minds of those present as to the desirability of securing one from the Q.V.J.I., of the Scotch branch of which association Princess Louise is President. Miss Guthrie Wright, the Secretary, explained at length the foundation and the aims of the Institute, and after various speakers had cordially expressed their interest in the present scheme, it was unanimously agreed to secure a Queen's Nurse for Peebles.

### ODD PENNIES.

Provost Rutherford proposed the institution of a "Sick Nurse Sunday" when he spoke at the annual meeting of the Kirkintilloch and Lenzie Nurse Society. He expressed the views held by many other persons when he said that numerous stray coins would in this way be collected in churches from people who would not otherwise like to offer such small gifts. The work of Miss Gilchrist is highly valued by the poor, and thoroughly appreciated by all who know her unflagging energy and special suitability for skilled and sympathetic nursing.

### NURSE AND SUPERINTENDENT.

A year ago the pressing need for skilled district nursing was realised by the people of Aberdeen, and a committee of ladies was formed to consider the matter. Eventually Miss Armstrong, a Queen's nurse, was secured, and she laboured for five months single-handed, although, by right of experience and appointment, she held the rank of "superintendent." However, there were no funds forthcoming at the time for the maintenance of more nurses, so Miss Armstrong devoted herself to most able personal superintendence of the sick poor. She nursed 75 cases, paying 1,341 visits before the end of October, when another Queen's nurse joined her. A house has now been taken, and we think it will not be long before prosperous folks at Aberdeen see that the best practical help they can give to their poorer brethren is by additions to the number of nurses, thus making Miss Armstrong's name of "Superintendent of Nurses" no longer an empty title.

### CASUALTIES AT CHICAGO.

Since the work in the "Fair" grounds began at Chicago there have been 4,000 cases of illness or injury requiring hospital treatment. This prepares us somewhat for the announcement in the 'Trained Nurse' "that there will be a morgue, an ambulance service, a corps of trained nurses, four hospitals of thirty-three beds each, four hospital boats to patrol the lagoon, which is three miles long, and sub-stations throughout the grounds for accident cases." Of course the accommodation is only to be temporary, and the sick or wounded will be promptly removed from the Fair either by the lake or by rail.

### SHORT ITEMS.

At the conclusion of one year's service on the private nursing staff of the Hampstead Home Hospital the nurses will in future receive 5 per cent. on their earnings.—A sale of work will be held in aid of the funds of the hospital on 12th and 13th May.—The inmates of the Beach Rocks Convalescent Home, at Sandgate, were much alarmed by the recent landslip, but a panic was avoided by the presence of mind of the nursing staff and the secretary, the latter remaining at the Home during the night.—There is an increasing demand for good mental nurses with general hospital training, and a few more can be taken on to the Co-operation, 8, New Cavendish Street. All other vacancies are now filled up there, and the next election of nurses will take place in November.—At Berwick-on-Tweed, Miss Law, the Queen's nurse, has won golden opinion from rich and poor. Work increased so rapidly, as shown by the first annual report, that the urgent necessity for a second nurse is universally acknowledged.

### THE DISABLED NURSE.

The "Disabled Nurse" writes in grateful acknowledgement of the weekly grant of ten shillings, and adds, "I am sorry I cannot tell you I gain much ground. The last fortnight I have been suffering from an attack of bronchitis, and coughing shakes my back and increases the pain very much."

### "THE HOSPITAL" ENDOWED BED.

Nurse Thomas has collected 5s., making total amount received £13 16s.

### International Nursing Congress.

### MRS. BEDFORD FENWICK'S POSITION.

With reference to previous notes on this question (*vide* The Hospital for December 3rd, 1892, January 28th, February 11th and 25th, and March 11th, 1893), our attention has been called to a statement which appeared in a nursing print last week.

If Mrs. Bedford Fenwick is content to rest her case on the fact that the chairman of the Chicago Congress reports (1) he believes that when in Chicago in October last "Mrs. Fenwick suggested the formation of an International Congress," whilst ignoring his further statement that "The General Committee decided that a separate Nursing Congress was not expedient," and, further (2), "that Mrs. Fenwick was asked by Mrs. Flower to be chairman of the British section," whilst ignoring the statement of the chairman that he and his committee "know nothing of any such office," we are content to let matters rest there. We will only further point out that up to this date Mrs. J. M. Flower has not publicly confirmed or contradicted the statement attributed to her in this matter. Another statement made in this connection is contrary to the documentary evidence contained in the minute book of the Sectional Committee of the Hospitals Association, of which Mrs. Bedford Fenwick was chairman. Those minutes record and prove that the scheme for registering nurses did not originate with Mr. Burdett at all, but with a very able lady, Miss Catherine J. Wood, who has done more than any other person to make the registration of nurses a practical possibility. Mr. Burdett is not entitled to the credit or discredit of the proposals to establish the registration of nurses, as they did not originate with him, but were prepared and formulated by a committee of three ladies, of whom Miss Wood was the active spirit. It may be interesting to add that Mrs. Bedford Fenwick was not a member of this committee of three.

### Management of Consumption.

### VII.—PNEUMOTHORAX AND ULCERATION OF LARYNX.

Pneumothorax, or the presence of air in the pleural cavity, is one of the modes of death from consumption. It has been calculated that about 5 per cent. of deaths from consumption are due to this cause. Since it is a mode of death it is not surprising that almost every case of pneumothorax in connection with consumption proves fatal. Occasionally it occurs early in the disease, and patients live some considerable time after its occurrence ; but in all cases life is shortened.

Pneumothorax is produced in the following way. By the breaking down of deposits of tuberculous matter in the lung a cavity is formed. The pleura covering the lung is always chronically inflamed and thickened in consumption, and is more friable than in health. Now, if this cavity is close to the surface of the lung, during violent fits of coughing or periods of undue exertion, it is very liable to rupture into the pleural cavity, and when this does occur, the air taken into the lung is expelled into the pleural cavity, together with the contents of the cavity which has just ruptured. It more often happens that owing to the inflammation of the pleura, the pleural cavity is obliterated in parts, and if this untoward event occurs, no air escapes into the pleural cavity, for the simple reason that there is no cavity for the air to escape into.

The contents of the excavation which escape with the air into the pleural cavity, rapidly set up an acute inflammation of the pleura, and very soon pus is formed, and the complication is then termed pyo-pneumothorax. The symptoms of the occurence of pneumothorax are somewhat as follows: The patient, after some movement attended with exertion, or after a fit of coughing, suddenly gasps for breath ; he at the same time feels a sensation in one side, as if cold air or water was being poured down the inside of the chest. There may be an acute pain in the side, which is generally described as of a stabbing or rending character. The respiration may amount to 60 in the minute, so great is the dyspnœa, and the pulse rises in proportion. There is often blueness of the lips and finger ends. If now the chest be looked at, it will be seen that one side is more bulged than the other, and that that side which is bulged moves scarcely at all during respiration. The heart is generally displaced to the opposite side of the pneumothorax. If the temperature be now taken, instead of being perhaps elevated, it will be found to be extremely low—96 or 97 deg. The shock of the pneumothorax has produced this lowness of temperature. Very soon though, perhaps in six hours, reaction sets in ; and this, together with the inflammation of the pleura, combines to send the temperature up higher than it was before. At the onset of the attack the voice becomes whispering, and the hands and feet cold. Such, then, are the main features of an attack of pneumothorax. The patient may succumb very quickly, or may linger on for weeks or even months.

In nursing a case of pneumothorax, the first thing to be done is to send at once for the medical attendant, as he may sometimes be able to avert an immediately fatal termination. The patient should be placed in bed, and covered with warm blankets, hot bottles should be applied to the feet, and a small quantity of brandy allowed.

If the pain is excessive, poultices may be applied to the side, and, as a rule, they give relief. Much often depends upon the promptitude of the nurse in sending for the medical man. Lives have been saved by the timely insertion of an aspirating needle, and freeing the pleural cavity of the air. And this is why it is so important to recognise what has happened. It may be said that sudden dyspnœa, attended with rapid fall of temperature and pain in one side or other, in a case of consumption, suggests pneumothorax.

Treatment generally consists in stimulants, such as ether and ammonia ; or sedatives, such as morphia and chloroform.

*Ulceration of the Larynx.*—Consumption is frequently attended with tubercular disease of the larynx, and when this does occur it renders the case most distressing to nurse. When a person is affected with this disease, which is styled laryngeal consumption, his voice becomes hoarse ; it pains him to talk, to swallow, and to cough—eventually, as the disease progresses, his voice becomes reduced to a hoarse whisper ; his countenance wears a pained, distressed expression ; all attempts to take food being attended with pain ; he rapidly emaciates, and is practically worn out by suffering and want of food ; he is unable to expel from his chest the phlegm, and in the last stages of the disease the back of the throat may become involved in the ulcerating process.

If one looks at the larynx (or organ of voice) with a laryngoscope, it can be seen that parts of the organ are swollen, and that on and around the vocal chords are small ulcers. These small ulcers are tubercles which have broken down. It is very rare to find tubercles in the larynx without at the same time finding also tubercles in the lung.

The nurse's efforts in this class of case must be directed to feeding the patient and easing his pain. It will be found that all hot foods irritate the throat, and great care should be taken not to allow lumpy solid food, as it is often impossible for the patient to swallow it, and when he does he runs the risk of choking. The throat just before taking food may be rendered tolerably insensitive by giving the patient small lumps of ice to suck, or cocaine 2 per cent. solution may be sprayed on to the throat; in this way patients can often take their food without any great discomfort.

The pain in the throat may also be checked by wet compresses continually applied to the outside of the throat.

Extreme cases of laryngeal consumption are only met with at the end of the disease, and all treatment at best is only palliative.

### A Nurse's Life in Canada.

By a Canadian Lady Superintendent.

English friends ask what my life is like now that I have really entered upon hospital work in British Columbia, and I will try and answer their questions as far as I can. I will draw aside the curtain for a little bit, so that all may take a peep for themselves.

Imagine, then, when sleeping comfortably and peaceably on a nice little iron bedstead on a December morning, being suddenly aroused to half consciousness by the sound of a large bell. One dreams for a moment of fire, and then, as the bell keeps on ringing, one really awakens, to find that it is the nurses' "rising-bell." Although six o'clock, it is dark as midnight, but we get up quickly, for we know that we must not only be ready ourselves, but the beds must be made and the room in order before half-past six, when breakfast will be on the table. At seven o'clock, another bell gives warning of prayer-time, and all repair to the parlour, when a hymn is sung and a few comforting words read from the Bible; a short prayer follows, and we find ourselves in the throng of white aprons wending our way towards the wards.

These are long rooms, each containing twenty beds—ten on each side—and most of them occupied. We enter a medical ward for female patients; some of them suffering from heart disease, others from rheumatism, some from typhoid fever, phthisis, &c. The night nurses have made the ward tidy, and the convalescent patients comfortable, as far as soap and water can do it. They are now giving their report to the head nurse, so I hasten to the bath-room, and return, armed with comb, brush, soap, and a wash bowl, containing warm water. I commence with the first patient on the south side of the ward, as I am a junior nurse. She is a little girl named Jenny, and has typhoid fever, and would get on nicely if she did not fret so much. Poor little girl! no wonder she is anxious, for her mother is dangerously ill with the same disease and now occupies a bed on the other side of the ward, while Isaac, her big brother, is a patient in a male ward, Jenny looks up to greet me with a feeble little smile, and her first question "How do you think mother is this morning, nurse?" Makes my heart go out to the little one. I bathe her hands and face, and comb her hair, talking quietly to her all the while of Santa Claus and his Christmas visits to the hospital. When she is made trim and neat, I leave her smiling and happy, and pass on to the next bed. I cannot tell of all the the patients for this would take too long, so I must first mention that while I have been getting the patients on the south side of the ward washed and combed, another nurse has been equally busy on the north side, therefore everybody is now ready for breakfast, which has been served by a third nurse, and is being carried in on trays by patients so far convalescent as to give a helping hand in some of the lighter work.

After breakfast is over, and the maid has done the sweeping, the ward has to be set in order, so I proceed to wipe the dust off the stands and other furniture with a damp cloth, straighten the quilts, and see that every bed stands in line. Before doing this I have rubbed the backs and bony prominences of all the patients in bed, for we have to be very careful lest our patients develop bed-sores. Our superintendent is fond of saying, "I hope my nurses will never see anything in the way of bed-sores except imported ones." And it is generally understood that in this particular good nursing always tells. So I try to be faithful, for I want to be able to say when I am through that there is no blot of this kind on my record.

Certainly, "rubbing backs" sounds an easy part of the work, and yet, even in his, a nurse can do well or ill. I was surprised when I first thought of this. Our superintendent mentioned it in class one day, and in order to impress it on our minds told us of a patient who once said to her, "Do you know there are two ways of rubbing backs? One is when you rub because it is time to rub, and the other is when you rub to make the patient comfortable!" I am so anxious to remember all these things that I believe I shall take for my motto, "The patients comfort always." I think now that everything is done, but in order to be quite sure I stand at the end of the ward and look along the row of beds, trying to see if I can find anything to criticise in my own work. Don't imagine for a moment, though, that during all this time there have been no interruptions. Oh, dear, no! Mrs. Jones has had a drink several times; Mrs. Brown, with rheumatism, has had to have her leg placed in a more comfortable position; poor Mary, with consumption, her pillows re-arranged, and the delirious patient at the end of the ward, who has been responding to some imaginary call upstairs, has frequently been prevented from jumping out of bed—all these and numberless other interruptions have occurred. It is now half-past nine, and the bath-room, which is my especial work, has still to be set in order.

We used to hear a great deal about the scrubbing the nurses had to do in hospitals. Well, here it consists of this: We scrub a board about two and a half feet long, and two feet wide, which usually lies on the top of the bath-tub, and is called a poultice-board. We do scrub that, and scrub it well, and it is always kept spotlessly white. Instead of feeling it a humiliation, we soon learn to take a great deal of pride in seeing how white we can keep it; then the bath-tub has to be made clean, and all the utensils, and a nurse needs to have a conscience when she sets a bath-room in order, especially when there are cases of typhoid fever in the ward. One of the first things to learn is that she must have everything thoroughly aseptic, which simply means so clean that it cannot be made cleaner. This is why these things are nurses and not maids' work. The latter do not understand the importance of them. My own duty being done, the maid comes to do the rest, while I proceed to the pantry and set a tray with mugs of beef-tea and a plate of biscuits. Whilst I am distributing this, the house doctor makes his morning rounds, attended by the head nurse, who always carries a small writing-pad, on which she puts down his orders for the patients. It must be very nice to be a head nurse, and wear a black band on one's cap, I think to myself.

At this moment a whistle sounds, and going to answer it, I find a new patient just admitted in the office, is to be sent up to our ward—"a stretcher case." I hasten to the cupboard, and get a long rubber sheet, turn back the bed-clothes, and spread it over the bed, none too soon, for the new patient is already arriving. She has been picked up in the street, and is paralysed. How shall I describe her, to make clear her appearance, as she lay there on the stretcher? Dirty beyond description—face, hair, body, and clothing—and such clothing, too! She seems to have borrowed skirts from all her friends, and tied them with many strings around her waist. I take off her shoes first, then her stockings, and with the assistance of another nurse, remove her other clothing, and send it out of the ward to the fumigating room. I get plenty of warm water, soap, a pair of scissors, and some ammonia, and set to work. It looks hopeless, I confess, but in half an hour she is clean, feet and all, and evidently quite proud of her transformed condition. During this time the senior nurse has been busy making poultices, giving out medicines, stimulants, &c., and looking after the comfort of the patients generally. The head nurse has re-written the orders for the day, so that the treatment for each patient may be seen by simply going to look at the slate kept for this purpose. As soon as a nurse has carried out an order she draws her pencil through the number indicating the hour when the treatment was due. She then records what has been done on a chart which hangs by the patient's bed. This chart is very useful, and the visiting physician never fails to look at it, for it not only tells him when medicines have been administered, poultices applied, &c., but keeps him informed as to the condition of the patient since his last visit. The temperature, pulse, and respiration are all noted, and vomiting, delirium, &c., recorded.

But it is now dinner-time, and my head nurse tells me I can go to dinner first and remain off duty for an hour afterwards. This hour off duty always seems a great boon. I usually go out for a brisk walk, or else read or rest. Recreation is not included in the time allowed for meals as is sometimes supposed. But I must describe the dining-room. There are several small tables in it, one for head nurses, another for seniors, one for juniors, and another for probationers. No one ever thinks of saying that long word though, but abbreviates it, and therefore a new nurse during the whole of her first month is called a "probe." We are not supposed to discuss hospital experiences, &c., during meal-time, but I fear we often forget this. When I returned to the ward, dinner had been served and cleared away, and it therefore fell to my lot to set the ward in order for the afternoon; the quilts and stands needed to be re-arranged, and many little wants attended to once more. The senior nurse has worked hard trying to save little Jenny's mother, but it is of no avail—she has just breathed her last, and little Jenny is now an orphan. Everything was done for this patient that loving hands could do, for nurses often grieve deeply when they lose a patient.

But what is this tramping of feet drawing nearer and nearer? The door opens, and one of the visiting physicians or professors enters with his class of medical students, twenty or thirty young men. They pass from bedside to bedside examining the various cases, the head nurse standing near, meanwhile, ready to render any assistance that may be required. This is called "a bedside clinic," and may be, and probably is, very nice and helpful to the students, but between ourselves it isn't a bit nice for the nurses, for it upsets everything. When four o'clock comes I go to the storeroom for our afternoon supplies, bread, tea, &c., and then on my return I begin folding the clean clothes on the large ward table. I do this seated, folding sheets first, making them all the same size, then towels, pillow-slips, &c., all in the same way, and placed in the cupboard in separate piles. At five o'clock tea is served, and custard and toast made for the convalescents. Tea over, the wards must be made tidy once more, the white quilts folded and taken off for the night. Backs must be rubbed again, beds freed from crumbs, wrinkles smoothed out of the sheets, and pillows turned. Also the candle, medicines, poultice-cloths, &c., put ready for the night nurse, and all patients left comfortable. When the night nurse arrives the head nurse hands her the book containing all the orders for the night, telling her at the same time about the new patients, and the various changes which have occurred during the day, and then she turns to me and says, "you may go off duty now, nurse." Yes, I'm off duty, and to-morrow I expect to have all the afternoon off and intend doing wonderful things in the way of shopping, &c. I have my lecture notes to re-write; but as I enjoy it so much, it really becomes a pleasure instead of seeming like work. I do work steadily, I admit; but I am always so hungry, and sleeping is really a pleasure. Everybody tells me I am looking so well. It is nearly ten o'clock, so I must hasten away, for that is the hour we begin to get ready for bed. People ask if I am happy; if I ever get home-sick. Yes, I am happy, truly happy, for I like my work. I try to remember when patients are fretful and peevish that they are sick, and consequently not responsible. As for being home-sick, I have not really time for that.

### Nursing in Portugal.

A momentous change is announced in the national hospitals of Portugal. It is one which does not seem to commend itself to either the doctors or the administrative staffs, who have not, apparently, been consulted on the matter. The Portuguese Government has, however, decided to banish the lay nurses from all the national hospitals, and to replace them by Sisters of Charity. We sincerely trust these ladies have had adequate training for the important profession of sick nursing, otherwise the change is not likely to benefit the patients, or to add to the efficiency of the management.

### Appointments.

[It is requested that successful candidates will send a copy of their applications and testimonials, with date of election, to The Editor, The Lodge, Porchester Square, W.]

Midland Counties Home for Incurables at Leamington.—Miss J. Brigham has been appointed Matron of this Home, where she had previously filled the post of Assistant Matron. Miss Brigham was trained at St. Bartholomew's Hospital, and we cordially congratulate her on her promotion.

Woodlands Convalescent Institution, Rawdon, Leeds.—Miss Alice Miller has received the appointment of Matron at the Woodlands Convalescent Institution. Miss Miller was trained at the Royal Southern Hospital, Liverpool, and was afterwards Night Superintendent at Bradford Infirmary for one year.

Worcester General Infirmary.—Miss Edith Bellars has been appointed Matron of Worcester General Infirmary. Miss Bellars was trained at the London Hospital, and afterwards held the post of Night Superintendent at the Bristol General Hospital for nearly five years, and subsequently that of Matron and Superintendent of Nurses at the Swansea General Hospital for more than three years. Such varied and valuable experience renders Miss Bellars specially suited for her present important appointment, and we heartily wish her success.

### Minor Appointments.

Bradford Infirmary.—Miss Katherine Mackenzie has been made Night Superintendent at Bradford Infirmary. Miss Mackenzie was trained at the Western Infirmary, Glasgow, and the Royal Hospital for Sick Children, Edinburgh. She afterwards held the posts of Sister and Assistant Matron at the Jenny Lind Infirmary, Norwich, and her testimonials are excellent.

### Hints for Nurses.

*The Practitioner** for March contains some very interesting matter, portions of which may well be commended to the special consideration of nurses. The article entitled, "The Effect on Sucklings of Purgatives Administered to the Mother," is contributed by Dr. William Gow, and it treats of facts with which all midwives and monthly nurses should be acquainted. To quote Dr. William Gow's own words, "There is a widespread impression, especially amongst monthly nurses, that purgatives administered to the mother often lead to disturbance of the bowels of the suckling infant. There seems, however, to be no accurate knowledge as to the frequency with which this occurs." Certainly, a perusal of the short article, which includes carefully-prepared tables, will furnish all readers with most "accurate knowledge." Mr. E. Owen's treatment of "Post Nasal Growths" hardly concerns nurses, except in one paragraph, which will cause consternation in some of our readers. "At the conclusion of the operation the child should be left upon the table without a pillow, or laid flat on the floor, with his face turned slightly over; and in this position he may be allowed to sleep off the effects of the chloroform. Thus, if he bleeds, 'hawks,' or vomits, he does not soil the bed." Why should a poor little child, any more than an adult, be left on the floor? Surely the nurse in charge of the unconscious patient can always avoid the soiling of the sheets by judicious arrangement of towels or mackintoshes. If she cannot do so she must be quite unfit to be trusted with the case. We venture to think that nurses in general would strongly oppose such heroic treatment of their little charges. It would even be better to sacrifice a sheet or two than to deprive the patients of the comparative comfort and safety of their warm beds, at any rate from the nursing point of view. "The Practitioner" also gives a short lecture by Dr. Ernest Sansom on "The Medical Examination of Children" which shows very clearly that the writer speaks by personal experience of the points requiring special attention in dealing with young patients. The close observation of every detail, the infinite patience and tact required for successful treatment may well be studied advantageously by the intelligent nurse. We do not recommend such articles as these to new probationers for they would assuredly only add to the difficulties of the recruit recently imported into a children's ward. Experienced nurses, however, may well add deeper studies to those nursing manuals which they have already mastered.

**The Practitioner*, a monthly journal, price 8d. Edited by Lauder Brunton, M.D., LL.D., F.R.C.P., F.R.S.; Donald Mac Alister, M.A., B.Sc., F.R.C.P.; I. Mitchell Bruce, M.A., M.D., F.R.C.P. Published by MacMillan and Co.

### Everybody's Opinion.

*[Correspondence on all subjects is invited, but we cannot in any way be responsible for the opinions expressed by our correspondents. No communications can be entertained if the name and address of the correspondent is not given, or unless one side of the paper only be written on.]*

### LINEN NIGHT-DRESSES.

"A. E. I." writes: I have been reading with great interest the articles on "Management of Consumption" appearing each week in THE HOSPITAL, and have gained much useful information from them. This week, however, I have been much surprised to see linen night-dresses recommended for persons troubled with night sweats. Can the writer, presumably a man, really mean linen? Louis C. Parkes, in his book on Hygiene, says, "Linen is a good conductor of heat, and a bad absorbent of moisture, like cotton, and is even an inferior material for underclothing." In Newsholme and Scott's "Domestic Hygiene" we read that "children and weakly adults should wear woollen under garments throughout the whole year, as they can now be obtained of any thickness to suit different seasons." Now, as linen is a good conductor of heat and a bad absorbent of moisture, it would surely have the effect of leaving the skin cold and wet, whereas flannel, being a non-conductor of heat and a good absorbent of moisture, would reverse this process, and leave the skin warm and dry, the latter being undoubtedly a condition of greater comfort and less danger to the patient. Of course, the flannel could be of the thinnest description, and the bed clothing as light as possible so as not to induce spurious sweating, but I cannot help thinking that most consumptive patients would strongly object to linen next to the skin night or day. However, we live and learn, so perhaps my opinions are all wrong. I should, however, greatly like to hear what other nurses think on this subject.

"A LADY EXPERIENCED IN NURSING" writes: Touching the wearing of linen by consumptive patients, I beg to submit one or two practical reasons against such clothing. I have often remarked the extreme sensitiveness of the skin of these persons, especially to external cold. During attacks of hæmorrhage free applications of ice are not objected to, but in the changing of sheets and garments warmth has always seemed to me essential, and the touch of a cold hand or material causes evident discomfort. For fifteen months I had charge of a case of consumption in the warm, dry climate of the Canary Isles, and although the thermometer is rarely below 70 degrees, my patient always wore pyjamas of flannelette and a very thin gauze vest, made loose and buttoning like a jacket next the skin. This absorbed all moisture, was easily washed, and was considered more comfortable than cotton or linen night shirts. As the exertion of raising the arms in any stage of the disease is unadvisable for patients, it should be avoided by using garments specially shaped for the wearer's convenience.

### SOOTHING SYRUPS.

"A LECTURER" writes: Can nothing be done to stop the indiscriminate use of soothing syrups by ignorant mothers? In the course of one of my lectures on nursing, I said that many soothing syrups contained laudanum, and therefore should never be given without medical advice. After the lecture the clergyman remarked, "I am glad you mentioned soothing syrups, because the women in this village use it to an enormous extent." Since then I have lectured at some fifteen or twenty villages, and have always condemned the use of soothing syrup. In every case someone has come forward and said that the consumption of them in the village was really fearful. The general shop-keepers own to making enormous profits on this medicine, so naturally they encourage its purchase. It is no unusual thing to hear the poor mothers say they "have used it for five or six children' with no bad results. I should like someone to inform me if all soothing syrups are hurtful, and also if they often affect infants' brains, or have fatal results. Perhaps readers of THE HOSPITAL can give me facts, which are harder to get than opinions.

### CO-OPERATION.

NURSE ELITA says: I have seen Nurse Olive's letter in THE HOSPITAL, and think possibly a long-cherished scheme of my own may now be carried out. I am thoroughly well acquainted with the nursing world of Birmingham from having myself nursed there for twelve years. I have long felt what an advantage a Central Home would be to nurses engaged in private work, as well as to doctors and the public in general. Capital would, of course, be required to open the "Home" and to start it. I have a good connection, a little capital, and experience in general management and housekeeping. If Nurse Olive knows of other nurses willing to co-operate, I am ready to discuss the subject further.

### Co-operation or Competition?

The Honorary Secretaries of the Royal British Nurses' Association write: "The comments on her Royal Highness the Princess Christian's scheme for enrolling a body of nurses for attendance on cases of cholera, which have appeared in some of your contemporaries in London and the provinces, have led to misapprehensions on the subject which might lead to confusion when the time for action arrives. We therefore venture to ask you to be so good as to make it known through your columns that her Royal Highness's scheme does not involve the assumption by the Royal British Nurses' Association of the functions of an institution for the supply of nurses to the public; that the special committee of the association does offer to act as a central means of communication between sanitary anthorities and nurses in all parts of the country who may desire to join in the common effort to meet the expected epidemic; that the suggestions relating to the provision which should be made for nurses by the local authorities in the matter of remuneration, and in other respects, were formulated in conference with the council of the Society of Medical Officers of Health, and have been issued to sanitary authorities only; and that it is understood that the expenses incurred in the employment of nurses will not only be chargeable to the rates, and therefore not be incident on individuals, but also will be payable direct to the institutions to which they may be affiliated, or to the nurses themselves, in cases in which they may happen to be unattached."

The question of the remuneration of these "enrolled" nurses was carefully omitted in the first enthusiastic appeal for recruits, and it sounds sadly prosaic now to hear that the rates are to cover the expenditure "incurred in the employment of nurses," also that the money is to be payable direct to the institutions to which nurses belong. This leaves things much in their original condition, and proves emphatically that the whole movement was premature and superfluous. Most local authorities are aware that they can apply in all emergencies to hospitals and nursing institutions which have already made such arrangements as previous experience proved wisest. Many country hospitals and infirmaries boldly make answer to this effect when questioned on the subject. At the present moment our unconquered foe influenza is again in our midst, making raids in the most unexpected quarters. Nurses are, therefore, busy, but we hear of no one shirking these cases, and yet a roll was never instituted with regard to an epidemic which is a comparatively new, as well as a dangerous and even fatal, one. Instead of asking individual nurses to pledge themselves to obey sudden calls, which may simply mean immediate desertion of one patient in favour of another; concerted action with all the existing institutions must be taken. In short, this is what the matter has now resolved itself into, and it is indeed a pity that Princess Christian's kindly interest was not originally secured for the larger scheme, which must prove the only practicable one. Most of us know what sorrow is caused to a private patient by a change of nurses, even when this is unavoidable, but still more when it is done for a whim. We heard of such a case recently; the first patient was dying slowly, of a painful disease, and the nurse did her duty so cheerfully and tenderly that the sufferer felt that the bitterness of death would be lessened for himself and for his family circle by the presence of that faithful woman. But fate interposed in the person of the Superintendent, and she suddenly superseded the nurse by another from the institution, an equally good worker doubtless, but still a stranger. The only explantion given to the patient's friends was that a more important personage had requested the special services of the first nurse. This is a sample of the kind of thing that might constantly occur in connection with the roll. In the latter case the nurse might be unattached to any institution, so things would be worse. Leaving her ordinary patient without a trained attendant, she must run off when summoned to a case of cholera, which might end fatally soon after her arrival. The quarantine necessarily enforced would, however, prevent her resuming her original duty until after the prescribed period of isolation. Then, perhaps, her services might no longer be required by the deserted sufferer.

### For Reading to the Sick.

### STEPPING STONES.

Most of us have a desire for a higher, better life than the one we live, only we are hindered frequently by little motives of which in our hearts we are ashamed, or we go with the multitude, not exactly to do evil, but certainly to countenance foolish unbecoming things, and have not strength of mind to make an effort and free ourselves from the sins and follies which weigh us down.

The poet says, "Men may rise, on stepping stones of their dead selves to higher things," and by God's help it is so. Who that has mastered self in a small thing has not found himself on higher ground and fitter to make another struggle? A slight patiently borne, a grievance passed over cheerfully, these mount us up when we hardly expect it. Then, again, there are the steps which our Heavenly Father has put ready for us in climbing the hill of life. These are prayer and reading our Bibles and the ordinances of religion, but we turn aside from them; we can get on just as well, we think, without them, and so, through perversity, we keep going round and round at the bottom of the mountain. It is so much easier to wander among the grass and flowers than to trouble ourselves with the rocks above, that we at last lose the power, and when we would climb find we cannot do so. We are not left to ourselves, however; our loving Father sees the feeble effort, and the wish which prompts it, and He puts stepping stones in our way, which we may use if we will; sickness, trials, afflictions of all sorts, if taken as coming from His hand, will be a ladder by which to climb. The mountaineer uses every help he can get which will lead him along the path he wishes to go. And we have a guide who knows the mountain well. Christ has gone before and cut out the steps for us, and we can tread with confidence in His blessed footprints. Yet more, He stretches out His powerful hand for us to grasp, and saves us from many a slip, and if we hold hard He never lets go, but points us ever onwards and upwards to the goal where we would be, so that as we go day by day along the "common round" we forget that we are rising, till at last we find ourselves unwittingly in the pure air of the mountain top.

And when we look back and see how carefully and tenderly we have been led, what perils we have escaped by God's mercy, we realise that we "can reach no higher than the Son of God, the perfect Head and Pattern of mankind, to live and die by." We can safely leave ourselves in His keeping, who has guided us "o'er moor and fens, o'er crag and torrent," and led our feet one step at the time into the paths of peace.

### Where to Go.

Home Nursing.—A course of six afternoon lectures will be given by Miss Catherine Wood at Fitzoy Temperance Hall, Little Portland Street, commencing on Friday, April 7th. Syllabus and tickets for the course, 10s. 6d., can be obtained of Miss C. J. Wood, 27, Percy Street, Tottenham Court Road.

The Free Exhibition of Pictures at St. Jude's, Commercial-street, Whitechapel, is open from 10 a.m. to 10 p.m. daily from March 22nd to April 9th. The exhibition contains many charming pictures, including some by Watts, Millais, Dicksee, and Sant.

Lecture at Trained Nurses' Club, 12, Buckingham-street, on 7.45 on March 24th.

### An Infallible Cough Mixture.

A House Surgeon's Story.

I had not been many weeks settled in the Bottlebury Hospital before I made the acquaintance of the Bailey family, the old Bailey as well as the young Baileys. There were three generations of them—the old Bailey, a thin, bent, groaning, bronchitic old fellow, neither use nor ornament; his son, and son's wife; and other effects in the way of children too numerous to mention. I calculated that there were about thirteen of them altogether, but the juvenile fry was such a numerous tribe that I may have under-estimated it. How they lived in the two rooms and a cupboard, of which the house consisted, was a mystery to me. The old man slept in the cupboard; I cannot violate the truth by calling the little pen a room. There was a turn-up bed in the living-room downstairs, where also the master of the house pursued his calling as a cobbler; and in the room over this, three beds left only a narrow passage between the walls and themselves.

The house was cottage-like in its proportions, and looked upon the main street which runs through that part of the town where filth and all that is disreputable most do congregate; and whence, I regret to say, a large proportion of those who came under my care at the Bottlebury Hospital were drawn. But, notwithstanding their surroundings, there was much to admire in Bailey junior. He was steady and industrious, and it was much in his favour that the bringing up of his numerous family had not driven him to seek the delusive and destructive distraction of the public-house, of which, as well as of pawnshops, there were several conveniently close at hand. He must have been sorely pressed at times to make ends meet; but when I visited the house during the illnesses of members of his household I always found him working quietly and patiently, and apparently little upset by the latest case of illness. Constant practice had no doubt taught him to take things philosophically, whilst the necessity of working hard to keep the pot boiling, left no time for regret, or opportunity for despair. He used to sit under the window on a low stool at a little bench, on which were the hammers and nails, and awls, and wax, and thread with which he earned his living. And whilst he gripped a dilapidated boot with his knees, to which the leather apron descended from his breast, he seemed oblivious to the babel of sound going on behind his back; and unless some particular outcry, which his wife was unable to quell, called for his interference, he worked away as if work was the only thing of which he was conscious.

It was the old Bailey, so named to distinguish him from his son, or, if the speaker wished to be more precise, "the old hand," whose acquaintance I made in the first instance. Years before I came to the hospital he had been an in-patient for scalds of the foot, caused by one of the children upsetting a kettle of boiling water. A cough, to which the old man was subject, troubled him while he was having the foot treated; medicine was prescribed for it, and one afternoon, when I was sitting to see the out-patients of a member of the staff who was unable to attend, old Bailey presented himself for a repetition of his cough medicine. I had not seen him before, and on looking over his paper I found that he had been regularly supplied with the favourite cough mixture for the last five years.

"You want some more cough medicine?" I began.

"Yes, yer honour, I do," and he coughed ominously, as if to show me that his want was real.

"You have had it a long time," I said, looking back over the record of his attendance. "Cannot you do without it at this time of the year, and you could come up again during the winter and have it then?" It was then the middle of August.

"No, yer honour, I couldn't. I couldn't live without it; I'd never get through the nights unless I had it by me to take a sup just when the cough plagues."

And he fixed me with a look half cunning, half suppliant. The mixture was sweet and syrupy, pleasant to take and soothing in its effects, and I more than suspected that old Bailey had simply got to like it, and that the taking it had become a habit, which he could not break.

I argued the point with him some time longer; but nothing could turn him from the belief that life without the cough mixture was not worth having. Accordingly I ordered him a repetition of the medicine, and he went away invoking heaven to pour showers of blessings on my head.

Soon afterwards I had to visit some of the younger Baileys, and every time I went, the old man was lavish in the encomiums he passed upon me for granting him his medicine. More than a year passed; old Bailey still came regularly for his cough medicine, and I was more or less continually in attendance on the rest of the family. Then the young Baileys seemed to recover, and to be in for a spell of good health. I frequently saw the mother as I made my round of the streets and alleys in the neighbourhood, and as we had by this time become good friends, she always gave me a respectful recognition as I passed.

I should perhaps explain that no patient is allowed to remain on the books of the Bottlebury Hospital more than three months. Each time the limit expired old Bailey got his recommendation "renewed," and was then supposed to be a fresh patient. He was so counted in the hospital statistics, so that for each year of his attendance he was counted four times; and, therefore, during those five years that he had attended, when I made his acquaintance, he had figured as twenty patients. It is said that the end often justifies the means, but the desire to present subscribers with a glowing report seems hardly a sufficient excuse for thus cooking the statistics of a charitable institution.

*(To be continued.)*

### Notes and Queries.

### Queries.

(81) *Almond Flour.*—In The Hospital, March 4th, almond flour is recommended for diabetics. I have tried in vain to obtain it in Liverpool, and shall be greatly obliged for an address.—*A. E. H.*

(82) *Address Wanted.*—Please insert address of the London Medical and Surgical Aid Society.—*Country Nurse.*

(83) *Chiropody.*—Information wanted.—*A Nurse.*

(84) *Patients' Heads*—What is best treatment when hydr. perchlor. and carbolic soap fail?—*Workhouse Nurse*

(85) *Training.*—I want to know of a home where I can get some training in surgical work.—*Private Nurse.*

(86) *Morning Training.*—Where can I get instruction in nursing children? Morning only.—*R. T. P.*

(87) *B. N. A.*—Nurse Erica "wants to know," &c.

(88) *Gauze.*—Can anyone give me directions for making iodoform guaze.—*A. B.*

(89) *Maternity.*—Where could I get employment as maternity nurse.—*A. D. T.*

### Answers.

(81) *Almond Flour (A. E. H.).*—Callard's Diabetic Foods at 146, New Oxford Street, London.

(82) *Address Wanted (Country Nurse).*—The Surgical Aid Society, Salisbury Square, Fleet Street; and The Medical Aid Society, 2, East India Avenue, London.

(83) *Chiropody (A Nurse).*—You had better write to some of the advertisers in the weekly ladies' papers, and ask for terms, &c.

(84) *Patients' Heads (Workhouse Nurse).*—Can you get permission to cut the hair and apply a "carbolic cap" for a night or two? Carbolic soap is very unreliable as regards strength, owing to the volatile nature of carbolic. The lotion is better, but you should get doctors' sanction as to the strength you use.

(85) *Training (Private Nurse).*—You had better advertise in The Hospital, stating length of your previous experience and training.

(86) *Morning Training (R. T. P.).*—See reply to 85, also write to Miss Coleman, 2, Maida Vale. It is difficult to get such partial training.

(87) *(B. N. A.).*—*Nurse Erica* must give an address, also her full name if she wishes for an answer.

(88) *Gauze (A. B.).*—Unless you are a qualified chemist you had better content yourself with buying your dressings from good makers.

(89) *Maternity (A. D. T.).*—You had better advertise. We suppose you have no general training certificate?

### The Book World for Women and Nurses.

[We invite Correspondence, Criticism, Enquiries, and Notes on Books likely to interest Women and Nurses. Address, Editor, THE HOSPITAL (Nurses' Book World), 428, Strand, W.C.]

THE TRANSACTIONS OF THE SEVENTH INTERNATIONAL CONGRESS OF HYGIENE AND DEMOGRAPHY, LONDON, AUGUST, 1891. Edited by C. E. SHELLEY, M.A., M.D. Published by Eyre and Spottiswoode. Price 2s. 6d. each volume.

The "Transactions of the Seventh International Congress of Hygiene and Demography," which took place in London, 1891, have just been issued in thirteen volumes. In section IV. we find much interesting matter under the heading, "Infancy, Childhood, and School Life." At the first meeting of this section Mr. Noble Smith combated the generally received idea that children might be left to "grow out" of their deficiencies, saying truly, "This idea is not in accordance with medical experience." Dr. Octavius Sturges made some valuable remarks on the physical indications of injurious schooling, which "are of many degrees, not all of them obvious to unskilled observers. . . . Sooner or later (and sometimes it is very late), it occurs to some one wiser than the rest that the child is not perverse, but ill." The statements of Dr. Sturges, which were supported and emphasised by Dr. Cheadle, should prove most helpful, not only to teachers, but to all persons responsible for the physical well-being of children. Dr. Shelly's tables and statistics as to "Epidemics in Schools" should interest parents particularly, though the latter will probably shake their heads dubiously over the assertion that "the day school is much more open to attack than is the boarding school"; but the facts given appear to afford no opening for doubting their accuracy. Under the heading, "Some of the Laws which Influence the Growth of the Child," Mr. Arbuthnot Lane gives a number of facts which ought to be familiar to women, whether nurses or not. He puts things so clearly and forcibly, that we are constrained to wish his paper were extended to double its present length. He concludes with a paragraph which we cannot do better than quote for our readers: "I would suggest that, to meet the rapidly progressive deterioration of the people, especially in and about the large towns, before a woman is permitted to engage in an occupation entailing the care, feeding, or supervision of infants or young children, or before she be allowed to marry, she shall be obliged by law to show that she possesses a satisfactory knowledge of the general physiology, diet, and hygiene of infancy. . . It seems reasonable also that those who are engaged in the education of youth in day schools or boarding schools should be thoroughly familiar with the physiology of the skeleton, and that the State should require from them some guarantee of the possession of this knowledge, which is only second in importance to that which should be possessed by those having the care of infants and very young children." When this dream of the future is realised, "infant mortality," preventable diseases, and deformed children will cease to be a reproach to our land. But who will live to see what will truly be the Golden Age?

### THE BOOK WORLD FOR WOMEN AND NURSES—*continued.*

The Strand Magazine continues its interesting studies of Hands, suggesting always Browning's words :—

" As like as a Hand to another Hand,"
Whoever said that foolish thing
Could not have studied to understand
The counsels of God in fashioning,
Out of the infinite love of His heart,
This Hand whose beauty I praise.

But not all these hands are beautiful. Some are even grotesque. Among the most remarkable are those of Carlyle, "stubborn, combative, mystical," and his wife. Hers is brave and practical, but gives little evidence in repose of the keen wit and restless energy of the owner. It is interesting to read of the cast of the Queen's hands which was sketched last month that " while the moulds were being made her Majesty removed all the rings from her fingers except the wedding ring. This she was most anxious should not come off. I was in considerable fear lest the moulding process might remove it."

### BOOKS ON COOKING.

A Handbook of Invald Cooking. By Miss Boland (The Century Co.).

We have just received an excellent work on invalid cooking from America. The want that existed until lately in literature of the kind can be felt no longer. Miss Boland's "Handbook of Invalid Cooking," covers the whole ground occupied by the subject. It is well arranged, being divided into separate parts, dealing with the chemistry of food and the digestion, recipes, serving, the feeding of children, district nursing, and bibliography. The chapters dealing with serving and district nursing offer invaluable hints to nurses, and will prove of the greatest service. Miss Boland is instructor of cooking in the Johns Hopkins Training School for Nurses, and a member of the American Public Health Association; she therefore speaks with the authority of experience, and deals with her subject in a most intelligent and interesting manner.

We are pleased to hear that another text book on " Cooking for Invalids " is promised to us. Miss Juliet Corson, of New York City, has undertaken to bring one out during the course of the World's Fair, at Chicago, and it is generally anticipated that it will prove particularly suitable for nurse training schools.

Talks in a Hospital. By a Hospital Nurse. Published by the Society for the Propagation of Christian Knowledge.

"Talks in a Hospital," by a Hospital Nurse, is hardly descriptive of the strictly religious manual before us. In the very sensible preface we find this seasonable reminder : " We must not forget, however, that a nurse's chief work is to minister to the bodily wants of the sick and suffering committed to her charge. It would be a sad mistake if our zeal for souls were to make us remiss in the more obvious duties of our calling." The author has compiled short chapters for reading to patients, and also a course of " Short Bible Readings." The little book contains, besides, nearly a dozen hymns selected from "Hymns Ancient and Modern," short private prayers for the patients, as well as morning and evening prayers for general ward use.

